# Challenges and strategies regarding anaesthetic management of twin pregnancy undergoing redo aortic valve replacement

**DOI:** 10.1016/j.ijscr.2024.110176

**Published:** 2024-08-14

**Authors:** Muhammad Saad Yousuf, Misbah Qurban Ali, Syed Shabbir Ahmed, Hamid Iqil Naqvi, Khalid Siddiqui, Khalid Samad

**Affiliations:** Department of Anaesthesiology, The Aga Khan University Hospital, P.O Box 3500, Stadium Road, Karachi 74800, Pakistan

**Keywords:** Anaesthetic management, Twin pregnancy, Ventricular fibrillation, Redo aortic valve replacement, Complete heart block

## Abstract

**Introduction and importance:**

Redo aortic valve replacement in twin pregnancy presents significant challenges because of the elevated risks for both maternal and fetal health. Mortality rates range from 12 % to 21 % in specialised centres, with previous cardiac surgeries further elevating the risk. Pregnancy complicates cardiac surgery, with fetal mortality rates as high as 16–33 %.

**Presentation of case:**

A 31-year-old woman, 15 weeks pregnant with twins and with a history of mechanical aortic valve replacement, presented with worsening breathlessness and grade III dyspnoea. Echocardiography revealed severe valve obstruction, necessitating redo-aortic valve replacement and posterior aortic root enlargement. Despite intraoperative challenges, including ventricular fibrillation and postoperative heart block, she underwent successful surgery and pacemaker implantation, with both mother and fetuses remaining stable.

**Discussion:**

Optimal timing of surgery is crucial, considering fetal developmental vulnerability in the first trimester and maternal cardiac workload in the third trimester. Second-trimester risks are comparable to non-pregnant patients. A limited understanding of fetal-placental perfusion during bypass necessitates cautious management strategies, with emerging techniques like pulsatile perfusion showing promise. Anaesthesia selection prioritises fetal safety while monitoring fetal distress during surgery remains challenging. To achieve successful outcomes for both mother and babies in a twin pregnancy undergoing a redo aortic valve replacement, careful timing, appropriate surgical techniques, and meticulous perioperative care are essential.

**Conclusion:**

A multidisciplinary approach is crucial for managing twin pregnancy following redo aortic valve surgery. Careful planning, close monitoring, and specialised surgical and anaesthetic techniques are key to minimising risks to both mother and fetus.

## Highlights

• Redo aortic valve replacement in twin pregnancy poses significant maternal-fetal risks.
• A 31-year-old woman, 15 weeks pregnant with twins, required redo valve surgery.
• Intraoperative challenges included ventricular fibrillation and postoperative heart block.
• Successful surgery and pacemaker implantation ensured stability for mother and fetuses.
• Multidisciplinary approach with careful planning and monitoring is essential.

## 1 Introduction

Approximately 1 % to 4 % of pregnancies are affected by cardiovascular disease [1]. This accounts for 10–15 % of maternal deaths [2], which makes it a significant concern. More women with heart diseases are living to childbearing age and wish to get pregnant thanks to better healthcare. Because of this, there has been an increase in the number of cases involving pregnancy-related cardiac problems. Pregnant women who have pre-existing valvular heart disease face specific difficulties since the physiological changes during gestation may worsen their condition [3]. Even though diagnosis, methods, and treatments have advanced, caring for these patients before or after giving birth can be complicated and often involves balancing between the mother's well-being and that of her baby. Valvular heart disease might be detected while one is expectant following symptoms manifestation thus requiring careful management. While drugs are usually used for treatment initially, surgery may be required if conservative measures fail [4]. This case report explores how anaesthetic care was provided for redo-aortic valve replacement during twin pregnancy and emphasises on the need for interdisciplinary collaboration among healthcare providers to achieve optimal outcomes for both mother and fetus.

## 2 Presentation of case

A 31-year-old woman, who is 15 weeks pregnant with twins, was previously diagnosed with congenital aortic stenosis and had a mechanical aortic valve replacement in 2004. Recently, she's been experiencing worsening breathlessness over the past two months, now reaching grade III dyspnoea when she exerts herself. This is her first pregnancy with twins. During antenatal check-ups, a grade 2 systolic murmur was detected. Echocardiography revealed a thickened annulus with pannus formation around the aortic valve, resulting in a narrowed valve area of 0.2 cm2, left ventricular outflow tract (LVOT) of 17.7 mm, and left ventricular hypertrophy.

Considering the high-risk nature of her pregnancy and her worsening heart condition, the cardiothoracic surgical team decided to perform a redo-aortic valve replacement along with enlarging the posterior aortic root. The obstetric team discussed the potential risks to the fetuses, including a 15 % chance of mortality during the heart-lung bypass.

During the surgery, standard monitors were used, and an 18-gauge cannula was placed in her right hand with local anaesthesia. As a precaution, defibrillator pads were applied before anaesthesia. An invasive arterial line was inserted using Seldinger's technique before induction. Rapid sequence induction was performed with the administration of intravenous (IV) fentanyl 250 μg, etomidate 14 mg, and rocuronium 80 mg. The airway was secured using a size 7.5 mm endotracheal tube (ETT), which was confirmed by end-tidal carbon dioxide measurement and chest auscultation. After induction, a Swan-Ganz catheter and a central venous pressure (CVP) monitor were inserted via her right internal jugular vein for hemodynamic monitoring and fluid management. An additional 20-gauge cannula was also placed in her right hand.

During the surgery, the patient experienced a ventricular fibrillation episode, which was quickly resolved with a 200-joule shock from a defibrillator. The aortic valve replacement was then carried out using a cardiopulmonary bypass for 235 min, with the aortic clamp in place for 180 min.

**Fig. 1:**
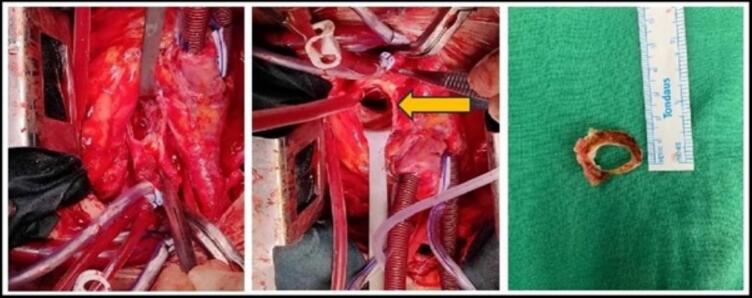
Pannus ring formation around the aortic valve.

The surgical team found a subaortic membrane causing severe obstruction in the left ventricular outflow tract, both below and at the valve level. To fix this, they replaced the aortic valve with a 21 mm mechanical valve and enlarged the posterior aortic root [Fig. 1]. Throughout the operation, non-pulsatile blood flow was maintained using a membrane oxygenator and blood cardioplegia. The patient's body temperature was lowered to 33 °C during the procedure and brought back to 37 °C when the cardiopulmonary bypass was discontinued. Anaesthesia was managed with a propofol infusion at 2 mg/kg/h and cisatracurium at 2 μg/kg/min.

The patient's mean arterial pressure was carefully kept between 50 and 80 mmHg during the cardiopulmonary bypass, and the pump flow rate was maintained at 4.6 l per minute. After the proximal grafting was done, the patient was gradually weaned off the cardiopulmonary bypass. Although one episode of ventricular fibrillation occurred post-bypass, it was successfully terminated with a 10-joule intracardiac shock by a defibrillator. Despite this, the patient continued to exhibit bradycardia rhythm, prompting the initiation of pacing at a VOO rate of 70 beats per minute throughout the remainder of the procedure and into the postoperative period.

After surgery, the obstetrician conducted a fetal examination to confirm viability. As no obvious heart sounds were detected, viability was confirmed using a portable ultrasound. The patient was moved to the cardiac intensive care unit for close monitoring. A careful anticoagulation management was done to balance the risk of thromboembolism with the safety of both the patient and the fetuses. Initially, intravenous unfractionated heparin (UFH) was administered to maintain therapeutic anticoagulation, with close monitoring of the activated partial thromboplastin time (aPTT). This approach allowed for rapid adjustments in response to any bleeding complications. As the patient stabilised, a transition to low molecular weight heparin (LMWH) was considered due to its safety profile during pregnancy. However, because of the mechanical valve's high thrombotic risk, the patient was ultimately transitioned to warfarin, with careful monitoring to maintain the international normalised ratio (INR) within the therapeutic range (2.5–3.5). This transition was managed in coordination with the maternal-fetal medicine team, balancing the need for effective anticoagulation with the minimisation of fetal risk.

The next day, extubation was done, but later the patient experienced a complete heart block. This prompted a consultation with the electrophysiology team. They began using a Holter monitor to track the patient's heart rhythm and eventually decided to implant a permanent dual-chamber pacemaker. The pacemaker surgery took place on the 12th day after the initial operation. Once transferred to a regular hospital room, the patient was able to go home on the 13th day after surgery. Table 1 summarises the timeline of clinical events and interventions.

**Table 1:** Timeline of clinical events and interventions.

Event	Description	Timeline
Initial presentation	Worsening breathlessness, grade III dyspnoea	At 15 weeks gestation
Antenatal check-up	Grade 2 systolic murmur detected; echocardiography performed	At 15 weeks gestation
Echocardiography findings	Thickened annulus, pannus formation, valve area 0.2 cm^2^, LVOT 17.7 mm, LV hypertrophy	At 15 weeks gestation
Preoperative multidisciplinary meeting	Discussion among cardiothoracic surgery, obstetrics, and anaesthesiology teams regarding surgical intervention	Prior to surgery
Surgical intervention	Redo-aortic valve replacement, posterior aortic root enlargement	Surgery Day (Day 1)
Intraoperative complications	Ventricular fibrillation (resolved with defibrillator shock)	During Surgery (Day 1)
Postoperative care	Monitoring in cardiac ICU, extubation, episode of complete heart block	Postoperative Period (Days 2–11)
Fetal monitoring	Fetal viability confirmed using ultrasound post-surgery	Immediate Postoperative Period (Day 1)
Electrophysiology consultation	Holter monitoring and decision to implant permanent dual-chamber pacemaker	Postoperative Period (Days 11–12)
Pacemaker implantation	Dual-chamber pacemaker implanted	Postoperative Period (Day 12)
Patient discharge	Discharged from the hospital	Postoperative Period (Day 13)

## 3 Discussion

Congenital heart disease is the most prevalent condition among pregnant women treated at referral centres, followed by rheumatic heart disease. The frequency of maternal heart disease during pregnancy is estimated to be between 0.3 % and 3.5 % [5]. Pregnancy is not usually associated with aortic stenosis, which is usually caused by a defective valve that is congenital. Mild cases usually don't pose significant issues, but moderate to severe cases can lead to heart failure and ischemia due to the heart's inability to adjust to increased preload changes and hypotension [6].

The timing of surgery is critical for fetal welfare, with the third trimester being a lower-risk period for the fetus but posing a higher risk for the mother due to increased cardiac output. Maternal mortality associated with such surgeries ranges from 1.5 % to 4.2 %, compared to fetal mortality rates of 9.5 % to 33 % [7]. Fetal survival to term after corrective surgery in the second or third trimesters has been reported. In this case, the patient's history of congenital aortic stenosis and previous aortic valve replacement compounded the complexity of the procedure, particularly given the advanced stage of her pregnancy.

Anaesthetic agents generally have minimal teratogenic effects, and vasoconstrictors are avoided due to their impact on uterine blood flow. Cardiopulmonary bypass (CPB) poses risks including coagulation changes, altered blood component function, vasoactive substance release, and hypothermia [8]. CPB during pregnancy can affect the delicate balance between the fetus and placenta, with reports of fetal bradycardia at the onset of CPB. Deep Hypothermia and rewarming during CPB can induce fetal hypoxia and pose potential effects on placental blood flow, while factors like haemodilution and uterine arterial spasm may contribute to placental perfusion alterations [9]. Effective anxiolysis, analgesia, and anaesthesia are crucial to prevent further cardiac function derangement during surgery and labour.

A multidisciplinary team determines the urgency of the operation based on the severity of the valve disease and the stage of pregnancy, with a primary focus on the safety of the mother and the babies. During the procedure, precautions were taken to minimise risks to the fetuses, and close monitoring was maintained to address any complications promptly. Post-surgery, ongoing assessment ensures optimal outcomes for both the mother's cardiac function and the well-being of the fetuses, highlighting the importance of comprehensive care and collaboration in managing complex medical scenarios. To address these risks, several precautions were taken during the procedure. Defibrillator pads were applied as a precaution against irregular heart rhythms. The viability of the fetus was checked both before and after the surgery. Temporary pacemaker support was used to maintain sufficient blood flow and prevent low blood volume and slow heart rate. The average normal haemoglobin>10 g/dl was maintained throughout the stay [10,11]. Corrective measures were confirmed using intraoperative transesophageal echocardiography (TEE) compared to the pre-incision assessment. To prevent inadequate blood flow to the uterus and placenta, high doses of drugs that constrict blood vessels were avoided. The volume of the bypass circuit was minimised through retrograde arterial autologous priming to decrease the risk of complications. To optimise blood flow to the uterus and placenta, mild hypothermia, and high-flow cardiopulmonary bypass was used, as severe hypothermia can cause fetal slow heart rate and premature labour [11]. Blood flow was maintained at 4.6 l per minute per square meter of body surface area while ensuring a mean arterial pressure of >70 mmHg. Tranexamic acid, a drug that reduces bleeding, was not used unless there were specific concerns about bleeding due to the natural tendency for increased blood clotting during pregnancy. To protect the safety and well-being of both the mother and the fetus, the management before, during, and after surgery was guided by careful consideration of their respective physiologies.

## 4 Conclusion

A well-coordinated multidisciplinary approach is crucial for managing a complex twin pregnancy after redo aortic valve surgery. Collaboration between cardiologists, obstetricians, and anaesthesiologists facilitates thorough planning and vigilant monitoring. Specialised surgical and anaesthetic techniques are essential for minimising fetal risks and ensuring maternal safety. Additionally, effective anticoagulation management, rigorous fetal monitoring, psychological support, and comprehensive postoperative follow-up are key to optimising outcomes for both the mother and her twins.

This study has been reported according to the SCARE criteria [12].

## Ethical approval

The study was exempted from ethical approval by the Ethical Review Committee at Aga Khan University in line with the institution's standard operating policy requiring only informed consent and not an ERC approval for case reports.

## Funding

None.

## Author contribution

**Muhammad Saad Yousuf** - concept, design, definition of intellectual content, literature search, manuscript preparation, manuscript editing, and manuscript review.

**Misbah Qurban Ali** - literature search, clinical studies, data acquisition, manuscript preparation, and manuscript editing.

**Syed Shabbir Ahmed** - clinical studies, experimental studies, data acquisition, manuscript preparation, manuscript editing, and manuscript review.

**Hamid Iqil Naqvi** - manuscript preparation, manuscript editing, and manuscript review.

**Khalid Siddiqui** - manuscript preparation, manuscript editing, and manuscript review.

**Khalid Samad** - concept, manuscript preparation, manuscript editing, and manuscript review.

## Guarantor

Muhammad Saad Yousuf

## Research registration number

NA.

## Conflict of interest statement

Nothing to declare.
